# SPECT/CT with ^99m^Tc-sestamibi for the evaluation of skeletal muscle perfusion after electrical muscle stimulation in athletes

**DOI:** 10.1590/0100-3984.2018.0006

**Published:** 2019

**Authors:** Beatriz Birelli, Mauricio Oliveira, Allan de Oliveira Santos, Willians Manso, Andreia Vicente, Elba Etchebehere

**Affiliations:** 1 Division of Nuclear Medicine, Campinas State University, Campinas, SP, Brazil.; 2 Brazilian Confederation of Aquatic Sports (CBDA), São Paulo, SP, Brazil.; 3 Nuclear Medicine and PET/CT, Sírio-Libanês Hospital, São Paulo, SP, Brazil.; 4 Medecell do Brasil, São Paulo, SP, Brazil.

**Keywords:** Electrical stimulation, Tomography, emission-computed, single-photon/methods, Tomography, X-ray computed/methods, Technetium Tc 99m sestamibi, Athletes, Muscle, skeletal/blood supply., Estimulação elétrica, Tomografia computadorizada de emissão de fóton único/métodos, Tomografia computadorizada/métodos, Tecnécio Tc 99m sestamibi, Atletas, Músculo esquelético/irrigação sanguínea

## Abstract

**Objective:**

The purpose of this study was to evaluate the effects of electrical muscle stimulation (EMS) on muscles, using ^99m^Tc-sestamibi SPECT/CT.

**Materials and Methods:**

We prospectively enrolled 20 consecutive male professional water polo players. The mean age was 25 years (range, 18-36 years). All athletes underwent ^99m^Tc-sestamibi SPECT/CT of the thigh (rectus femoris and vastus medialis muscle groups) before and after EMS. Images were quantified to identify increases in perfusion after EMS.

**Results:**

Before EMS, there were no significant differences between the right and left thigh (rectus femoris and vastus medialis muscles) in terms of perfusion (*p* = 0.4). However, the comparison between the pre- and post-EMS analyses of the same muscle groups showed significant differences in radiotracer uptake (*p* < 0.001), with a mean increase in perfusion of 128% for the rectus femoris muscle group (95% CI: 0.86-1.61) and 118% for the vastus medialis muscle group (95% CI: 0.96-1.79).

**Conclusion:**

^99m^Tc-sestamibi SPECT/CT is an objective means of evaluating blood flow in muscles submitted to EMS, which appears to promote significant increases in such blood flow.

## Abstract (pt)

**Objetivo:**

O objetivo deste estudo foi avaliar o efeito da estimulação elétrica transcutânea na musculatura esquelética utilizando-se SPECT/CT com ^99m^Tc- sestamibi.

**Materiais e Métodos:**

Foram prospectivamente avaliados 20 atletas masculinos de polo aquático (18-36 anos de idade; média de 25 anos). Todos os atletas foram submetidos a SPECT/CT com ^99m^Tc- sestamibi das coxas antes e após estimulação elétrica transcutânea dos grupos musculares reto femoral e vasto medial. As imagens foram quantificadas para identificar aumento da perfusão.

**Resultados:**

A comparação da perfusão dos grupos musculares (reto femoral e vasto medial) direito *versus* esquerdo durante o repouso não demonstrou diferença significativa (*p* = 0,4). Entretanto, quando comparados esses mesmos grupos musculares antes e após a estimulação elétrica, observou-se diferença significativa na captação do traçador (*p* < 0,001), com aumento perfusional médio de 128% do grupo reto femoral (IC 95%: 0,86-1,61) e 118% do grupo vasto medial (IC 95%: 0,96-1,79) no grupo estimulado.

**Conclusão:**

A SPECT/CT com ^99m^Tc- sestamibi é capaz de demonstrar de forma objetiva que a estimulação elétrica transcutânea aumenta significativamente o fluxo sanguíneo muscular.

## INTRODUCTION

Electrical muscle stimulation (EMS), also known as neuromuscular electrical stimulation or electromyostimulation, is a well-established therapeutic and physical conditioning procedure in sports^(^^1^^)^. Different patterns of stimulation have various biological effects, including the control of hyperalgesia^(^^2^^)^; hypertrophy of normal and denervated muscles^(^^3^^)^; and an increase in muscle strength^(^^4^^)^. At a frequency of 7-10 Hz, EMS promotes capillary vasodilatation^(^^5^^)^, which improves muscle metabolism and removes potentially harmful metabolites, thus accelerating muscle recovery^(^^6^^,^^7^^)^.

Although the use of EMS has increased in recent years, to promote recovery from exercise, it is applied empirically by sports physiologists on high-performance athletes^(^^8^^)^. However, the mechanisms of action and effects of EMS on nerves and muscles has not been elucidated, constituting a promising field of research.

The use of diagnostic functional imaging may demonstrate the increase in perfusion induced by EMS, helping validate its use. Nuclear medicine studies can use ^99m^Tc-sestamibi-a radiotracer that is taken up (by passive diffusion) in cells with high metabolism and mitochondrial content-to detect and quantify blood flow and metabolic activity. Although ^99m^Tc-sestamibi is commonly used in order to identify tissues with high cellular metabolism^(^^9^^,^^10^^)^, especially diseased myocardial tissue, it also can be used in the evaluation of certain types of cancer^(^^11^^,^^12^^)^. New hybrid technologies that allow the fusion of metabolic and anatomic images, namely single-photon emission computed tomography/computed tomography (SPECT/CT), can precisely locate and quantify the concentration of ^99m^Tc-sestamibi.

Whereas myocardial imaging with ^99m^Tc-sestamibi has been studied extensively, there have been only a few studies related to metabolic imaging of skeletal muscle. Because ^99m^Tc-sestamibi is taken up by viable myocytes, is retained within the mitochondrial membrane by electrostatic interactions, and has a half-life of 6 h, it is ideal for detecting altered muscle perfusion.

The purpose of this study was to use ^99m^Tc-sestamibi SPECT/CT to evaluate the effects of EMS on muscles. We hypothesized that ^99m^Tc-sestamibi SPECT/CT would demonstrate an increase in blood flow after EMS. To detect that phenomenon, we quantified increases in blood flow and determined which muscle groups show increased perfusion after EMS. To our knowledge, this is the first study using ^99m^Tc-sestamibi SPECT/CT to look for correlations between EMS and muscle activity.

## MATERIALS AND METHODS

This was a prospective study in which we enrolled healthy male professional (high-performance) water polo players, all of whom were over 18 years of age. Volunteers with claustrophobia, recent thigh trauma, or other limb lesions were excluded from the study. The study was approved by the local institutional review board (CAEE No. 31199714.0.9999.5461). All participants gave written informed consent.

All participants underwent ^99m^Tc-sestamibi SPECT/CT images at two time points: before and after EMS of the right thigh muscles. EMS was performed only on the muscles of the right thigh in order to compare the muscle metabolism in the stimulated (right thigh) muscles with that observed in the unstimulated (left thigh) muscles.

### EMS

Using a portable digital electric stimulator (Medecell, São Paulo, Brazil), we performed EMS of the rectus femoris and vastus medialis muscles of the right thigh for 25 min. High-frequency asymmetric biphasic pulses were applied. The EMS electrode locations and the distance between electrodes were similar for all participants. No electrodes were placed on the control thigh.

**Figure 1:**
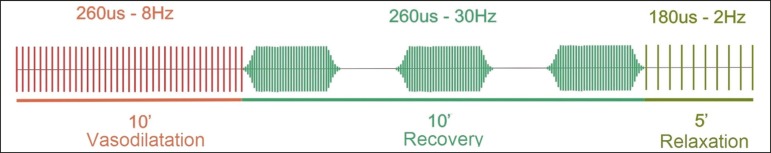
Three-phase EMS protocol.

The EMS was conducted in three phases (Figure 1). In the first phase, which promotes vasodilatation and improves blood flow, the EMS was delivered at 8 Hz, with a pulse width of 260 µs, for 10 min. The second phase increases blood glucose levels and induces active muscle recovery without recruiting type 1 fibers, which are normally responsible for resistance with high energy consumption and glycogen storage, thereby delaying muscle recovery^(^^13^^)^. In the second phase, which also lasted for 10 min, the same pulse width (260 µs) was maintained but the frequency was increased to 30 Hz. The third and final phase promotes muscle relaxation and endorphin release via afferent nerve stimulation, resulting in an analgesic effect and contributing to overall psychological improvement^(^^13^^)^. In the third phase, which lasted for only 5 min, the EMS was delivered at 2 Hz, with a pulse width of 180 µs.

### ^99m^Tc-sestamibi SPECT/CT

All participants underwent ^99m^Tc-sestamibi SPECT/CT of the right thigh (rectus femoris and vastus medialis muscle groups), before and after EMS. The pre-EMS image acquisition began 15 min after the injection of 370 MBq (10 mCi) of ^99m^Tc-sestamibi. The participants were submitted to EMS of the right thigh muscles, receiving an additional 1110 MBq (30 mCi) of ^99m^Tc-sestamibi during the procedure.

The SPECT/CT images of the thighs were acquired before EMS-15 min after injection of ^99m^Tc-sestamibi-and after the end of the EMS protocol. The images were acquired with a dual-head 16-channel SPECT/CT system (Symbia; Siemens Healthcare, Erlangen, Germany). The SPECT images were acquired with high-resolution collimators and a 128 × 128 matrix, at 40 s per projection. The CT images were acquired at 130 kV and 15 mAs, with a rotation speed of 0.8 s and a slice thickness of 5 mm. We performed iterative reconstruction of the images. The total mean effective dose per administered unit of activity applied per participant was approximately 3.2 mSv/MBq. We applied the "as low as reasonably achievable" principle. Because the dosimetry of ^99m^Tc-sestamibi is mainly to the gall bladder and not to the gonads, there is no significant effect regarding radiation exposure of the gonads due to the radiopharmaceutical. All of the participants were submitted to CT without gonad shielding because it would limit the region of coverage. Therefore, in order to reduce gonadal exposure, all participants underwent low-dose CT.

### SPECT/CT image quantification

**Figure 2:**
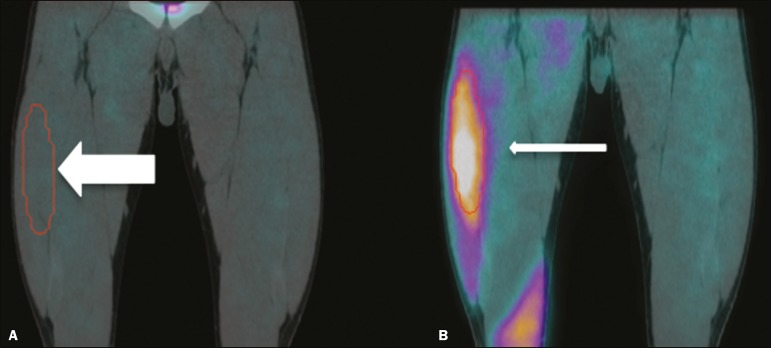
Images acquired before and after EMS (**A** and **B**, respectively). Note the increased perfusion in the rectus femoris and vastus medialis muscle groups after EMS. For quantification, a VOI, based on anatomical findings, was drawn around the rectus femoris muscle of the right thigh (thick arrow), and the same VOI was automatically copied for the same area after stimulation (thin arrow). A comparable VOI was subsequently drawn around the vastus medialis muscle (VOI not shown).

The radioactivity (^99m^Tc-sestamibi uptake) of the selected muscle groups after EMS was measured to identify any increase in perfusion. Voxel-based regions of interest, known as volumes of interest (VOIs), were manually drawn on the coronal images of the rectus femoris and vastus medialis muscles of both thighs before and after EMS (Figure 2). A voxel is the lowest element of three-dimensional medical imaging and is the basic unit for CT. The VOIs were delineated based on anatomical data from the CT scans.

Because the radiotracer dose after EMS was three times higher than the pre-EMS dose, the uptake in the control muscles would naturally be three times higher. Therefore, to compare the two thighs, the ratio between the right and left thighs in terms of the post-EMS/pre-EMS mean count ratios (post-EMS_ratio_/pre-EMS_ratio_ ratio) must be calculated on the basis of the following equations:

$$\begin{array}{c} \mathrm{pre}-{\mathrm{EMS}}_{\mathrm{ratio}}=\mathrm{mean}\;\mathrm{counts}\;\mathrm{of}\;\mathrm{the}\;\mathrm{right}\;\mathrm{muscle}\;\mathrm{groups}\\ \mathrm{before}\;\mathrm{EMS}/\mathrm{mean}\;\mathrm{counts}\;\mathrm{of}\;\mathrm{the}\;\mathrm{left}\;\mathrm{muscle}\;\mathrm{groups}\\ \mathrm{before}\;\mathrm{EMS} \end{array}$$

$$\begin{array}{c} \mathrm{post}-{\mathrm{EMS}}_{\mathrm{ratio}}=\mathrm{mean}\;\mathrm{counts}\;\mathrm{of}\;\mathrm{the}\;\mathrm{right}\;\mathrm{muscle}\;\mathrm{groups}\\ \mathrm{after}\;\mathrm{EMS}/\mathrm{mean}\;\mathrm{counts}\;\mathrm{of}\;\mathrm{the}\;\mathrm{left}\;\mathrm{mucle}\;\mathrm{groups}\\ \mathrm{after}\;/\mathrm{EMS} \end{array}$$

### Statistical analysis

The post-EMS_ratio_/pre-EMS_ratio_ ratios obtained for each muscle group were compared by using repeated-measures analysis of variance. The level of statistical significance was set at 5% (α = 0.05), and the analysis was performed with the Stata statistical software package, version 11.1 (Stata Corp., College Station, TX, USA).

## RESULTS

All 20 of the male professional water polo players completed the study. As can be seen in Table 1, the mean age was 25.1 ± 4.7 years (range, 19-36 years).

**Table 1:** Demographic characteristics of the 20 participants submitted to EMS and radiotracer uptake in both muscle groups during ^99m^Tc-sestamibi SPECT/ CT muscle perfusion scintigraphy, before and after EMS.

		Vastus medialis muscle group								Rectus femoris muscle group						
		Pre-EMS				Post-EMS				Pre-EMS				Post-EMS		
Athlete number	Age (years)	Right	Left	Right/left ratio		Right	Left	Right/left ratio		Right	Left	Right/left ratio		Right	Left	Right/left ratio
1	28	27	26	1.0		118	67	1.8		16	18	0.9		82	48	1.7
2	32	21	23	0.9		67	38	1.8		17	17	1.0		44	27	1.6
3	25	22	17	1.3		154	57	2.7		16	12	1.3		91	42	2.2
4	20	13	14	0.9		134	46	2.9		12	11	1.1		101	35	2.9
5	29	22	19	1.2		140	51	2.8		17	13	1.3		93	22	4.2
6	26	19	20	0.9		114	48	2.4		18	17	1.1		78	30	2.6
7	30	21	19	1.1		140	69	2.0		16	12	1.3		92	39	2.4
8	36	41	32	1.3		116	74	1.6		32	28	1.1		98	58	1.7
9	20	20	19	1.1		130	66	2.0		12	12	1.0		114	47	2.4
10	26	12	16	0.8		122	36	3.4		16	12	1.3		62	25	2.5
11	23	15	17	0.9		78	59	1.3		11	11	1.0		69	33	2.1
12	19	11	10	1.1		86	45	1.9		7	7	1.0		86	33	2.6
13	19	13	17	0.8		140	38	3.7		10	8	1.3		72	23	3.1
14	19	17	21	0.8		91	83	1.1		10	12	0.8		72	37	2.0
15	28	12	15	0.8		83	33	2.5		8	8	1.0		37	24	1.5
16	25	21	22	1.0		82	48	1.7		15	18	0.8		77	35	2.2
17	28	14	15	0.9		92	25	3.7		12	12	1.0		90	17	5.3
18	21	29	27	1.1		72	42	1.7		21	17	1.2		69	38	1.8
19	25	46	36	1.3		117	49	2.4		24	24	1.0		90	37	2.4
20	23	22	13	1.7		100	45	2.2		12	12	1.0		71	37	1.9
Mean	25.1	20.9	19.9	1.04		108.8	50.9	2.27		15.1	14.1	1.08		79.4	34.3	2.46
SD	4.7	9.2	6.4	0.23		26.2	14.9	0.7		5.8	5.2	0.16		18.5	9.9	0.91

**Figure 3:**
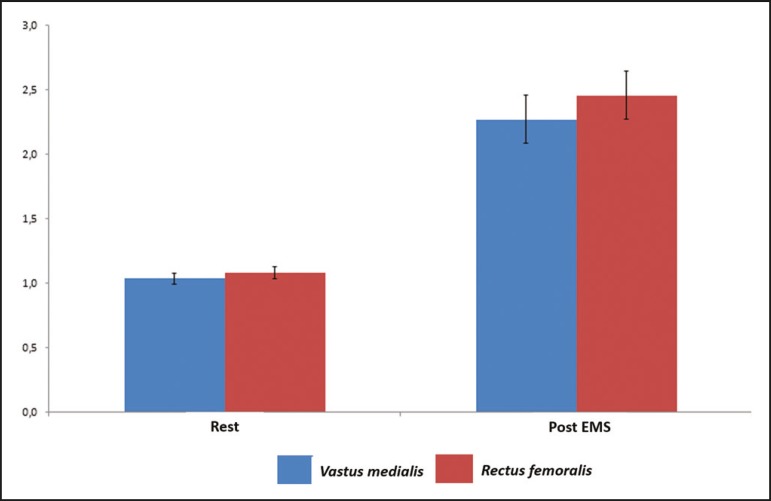
Radiotracer uptake values (means and ratios) for both muscle groups in the participants evaluated.

In the pre-EMS analysis, there was no significant difference between the right and left thighs in terms of the radioactive tracer uptake (as a measure of skeletal muscle perfusion) in the rectus femoris and vastus medialis muscle groups (*p* = 0.400). However, after EMS, there was a significant difference between the stimulated (right) thigh and the unstimulated (left) thigh, in terms of uptake (Figure 3). For both muscle groups (rectus femoris and vastus medialis), the post-EMS_ratio_ was higher than was the pre-EMS_ratio_, indicating a significant increase in perfusion (*p* < 0.001 for both). The mean increase in uptake was 128% for the rectus femoris muscle group and 118% for the vastus medialis muscle group (Table 2).

**Table 2:** Pre-EMS_ratio_ and post-EMS_ratio_ values obtained for both of the muscle groups evaluated.

Muscle group	Ratios	Mean	95% CI
	Pre-EMS_ratio_	1.04	0.95-1.13
Vastus medialis	Post-EMS_ratio_	2.27	1.90-2.65
	Post-EMS_ratio_ / Pre-EMS_ratio_	1.23	0.86-1.61
	Pre-EMS_ratio_	1.08	0.99-1.17
Rectus femoris	Post-EMS_ratio_	2.46	2.08-2.83
	Post-EMS_ratio_ / Pre-EMS_ratio_	1.38	0.96-1.79

## DISCUSSION

We have demonstrated that ^99m^Tc-sestamibi SPECT/CT scintigraphy can identify increased skeletal muscle perfusion after EMS. In addition, the evaluation of skeletal muscle perfusion with SPECT/CT scintigraphy allowed us to evaluate the specific muscle groups submitted to EMS (rectus femoris and vastus medialis) individually, as well as to perform a quantitative analysis. Notably, there was a two-fold increase in muscle perfusion after EMS.

There have been studies demonstrating that EMS alters skin temperature and increases blood flow^(^^14^^)^. However, to our knowledge, there have been no prior imaging studies investigating the consequences of EMS for the affected muscles. Theoretically, EMS performs selective muscle fiber stimulation, increasing muscle metabolism by increasing perfusion, thereby increasing energy reserves and promoting the removal of detrimental catabolites. In the present study, we successfully demonstrated that EMS does indeed produce this physiological effect (improving muscle metabolism by increasing perfusion).

Only a few studies have investigated the potential role of skeletal muscle perfusion imaging with ^99m^Tc-sestamibi. Such studies have focused on the assessment of peripheral vascular diseases, compartmental syndromes, uremic myopathy, statin-induced myopathy, systemic sclerosis, and Duchenne muscular dystrophy^(^^15^^)^. Only one prior study evaluated muscle response to neuromuscular electrical stimulation with ^99m^Tc-sestamibi scintigraphy^(^^16^^)^. However, none of those studies have focused on skeletal muscle perfusion using SPECT/CT imaging. The use of SPECT/CT provided accurate delimitation of the stimulated muscles, thus improving the semi-quantitative analyses in the present study.

Our study has some limitations. First, we evaluated a small number of participants, all of whom were male water polo players. Although that allows precise measurement and definition of the capability of the new EMS protocol to promote perfusion in this specific group, there is a need for further studies replicating our findings in other populations (e.g., women), in different sports modalities, and with shorter stimulation times. Shorter stimulation times might also result in a significant increase in muscle perfusion, thus allowing its application in reducing return-to-play intervals in a variety of sports. Another potential limitation is that we did not evaluate the clinical impact of the EMS procedure.

The increase in muscle perfusion promoted by EMS could be helpful for athletes and the general population, in various ways. By promoting muscle perfusion, it may be possible to increase the uptake and delivery of dietary supplements, antibiotics, and anti-inflammatory agents. If those effects are confirmed, EMS could also be used in order to increase the efficiency of drug delivery, thereby allowing the dosage of anti-inflammatory drugs to be reduced, thus lowering the risk of side effects; to increase the muscle uptake of standard doses, thus accelerating muscle recovery; to improve antibiotic delivery in patients with infection or arterial insufficiency; and to increase the efficacy of chemotherapy.

## CONCLUSION

EMS is a nonpharmacological, noninvasive, inexpensive, easily implemented method of improving muscle perfusion. Using noninvasive ^99m^Tc-sestamibi SPECT/CT scintigraphy, we were able to demonstrate, in an objective manner, that EMS results in a significant improvement in skeletal muscle perfusion.
